# Ultrasound-Guided Vascular Access Training: A Single-Center Experience Using Gelatin-Based Simulation Models

**DOI:** 10.7759/cureus.117123

**Published:** 2026-09-29

**Authors:** Sílvia Ferreira, Luís Miguel Afonso, Sofia Monteiro, Carolina Tintim Lobato, Ernestina Gomes

**Affiliations:** 1 Intensive Care Unit, Hospital Pedro Hispano, Unidade Local de Saúde de Matosinhos (ULSM), Matosinhos, PRT

**Keywords:** central venous access, critical care education, low-cost phantom, simulation-based training, ultrasound-guided access

## Abstract

Background: Ultrasound-guided central venous catheter (CVC) placement is increasingly recognized as the standard of care in intensive care settings. While it improves success rates and reduces complications, proficiency requires repeated practice, which may be difficult to achieve during clinical training. Low-cost simulation models provide a valuable alternative for skill development.

Methods: We created vascular access training models using gelatin, food coloring, and balloon materials. Five training sessions were conducted between January 2023 and January 2025 for physicians and residents working in an intensive care unit. Training sessions included lectures, ultrasound scanning of healthy volunteers, and hands-on ultrasound CVC puncture practice using gelatin models. Participants completed an anonymous online questionnaire assessing prior experience and satisfaction with the training.

Results: A total of 34 responses were collected. Most participants were specialty trainees in Internal Medicine, Anesthesiology, or Intensive Care Medicine. Despite limited experience with landmark-guided CVC placement among many trainees, nearly half had performed over 10 ultrasound-guided insertions, indicating prior exposure to the recommended technique. After the training, 97% reported increased confidence with the gelatin model, and 91% found volunteer-based ultrasound practice beneficial. The primary challenges identified were limited access to ultrasound machines and insufficient time for training.

Conclusion: Gelatin-based models are low-cost, accessible, and well accepted as tools for ultrasound-guided vascular access training. They may help skill acquisition and confidence, even among clinicians with previous experience, and are particularly suited for use in resource-limited settings.

## Introduction

The use of real-time ultrasound guidance for central venous catheter (CVC) placement is now widely recommended by scientific societies. It has become standard practice in intensive care and acute care settings, as it improves overall and first-pass success while reducing mechanical complications compared with landmark-based techniques [1-8].

However, achieving proficiency in this technique requires repeated practice, which presents challenges for the training of residents and physicians in real clinical settings [9,10]. In this context, simulation models ("phantoms") offer a safe and effective method for technical skill development, and simulation-based mastery learning has been associated with improved clinical outcomes and reduced complications during CVC insertion [9].

Various commercial simulation models exist for both peripheral and central vascular access training. However, their high cost often limits access, particularly in the context of medical residency training programs. Several low-cost alternatives to commercial phantoms have been described in the literature, including models using tofu, gelatin, psyllium, and ballistic gel, silicone tubing, or other tissue-mimicking materials [11-21].

The aim of this study is to present a practical, low-cost gelatin-based model for ultrasound-guided vascular access training (“phantom”) and to assess its perceived usefulness and feasibility in structured training sessions for ICU healthcare professionals. Between January 2023 and January 2025, structured training sessions were conducted for residents and senior medical staff in a Portuguese intensive care unit.

## Materials and methods

Participants were recruited through convenience sampling. At study initiation, all physicians permanently assigned to the intensive care unit were invited to participate. Thereafter, the sample was expanded on a rolling basis: at the start of each six-month rotation period, all physicians undertaking a clinical placement in the ICU during that period (i.e., residents/trainees rotating through the unit) were consecutively invited to participate. No random selection procedure was applied at any stage; enrolment was therefore based on the complete census of ICU-based physicians and of rotating trainees present in the unit during each recruitment window. 

Training models were created using gelatin, food coloring, and long latex balloons (see Appendix 1 for detailed instructions on model preparation and ultrasound equipment used; see Figures 1, 2). Knots were tied at intervals along each balloon to create multiple distinct segments, allowing trainees to practice on different sections of the simulated vessel. After puncturing the model, the same site could not be reused, as the needle track remained visible on ultrasound imaging.

**Figure 1:**
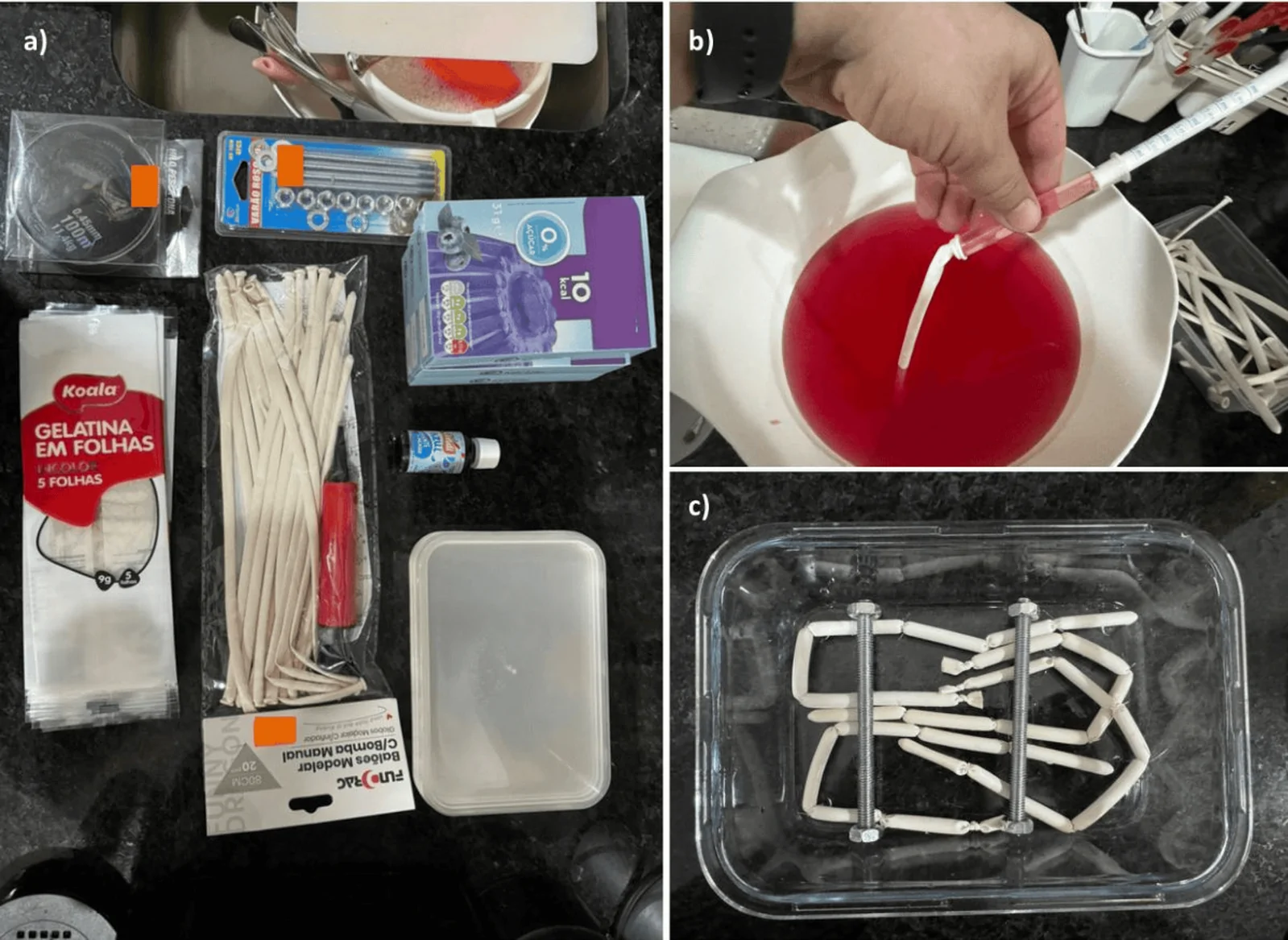
Preparation of a homemade ultrasound phantom a) Materials (tray, powdered and sheet gelatin, food coloring, cylindrical latex balloons, nylon thread, bolts with nuts) required for the preparation of the ultrasound training model. b) The cylindrical balloons were filled with water mixed with red food coloring to simulate blood (representing peripheral veins) and tied at intervals with nylon thread to allow for multiple puncture sites along each balloon. c) The filled balloons were arranged in the tray, secured with bolts to keep them submerged, and the tray was then filled with gelatin.

**Figure 2:**
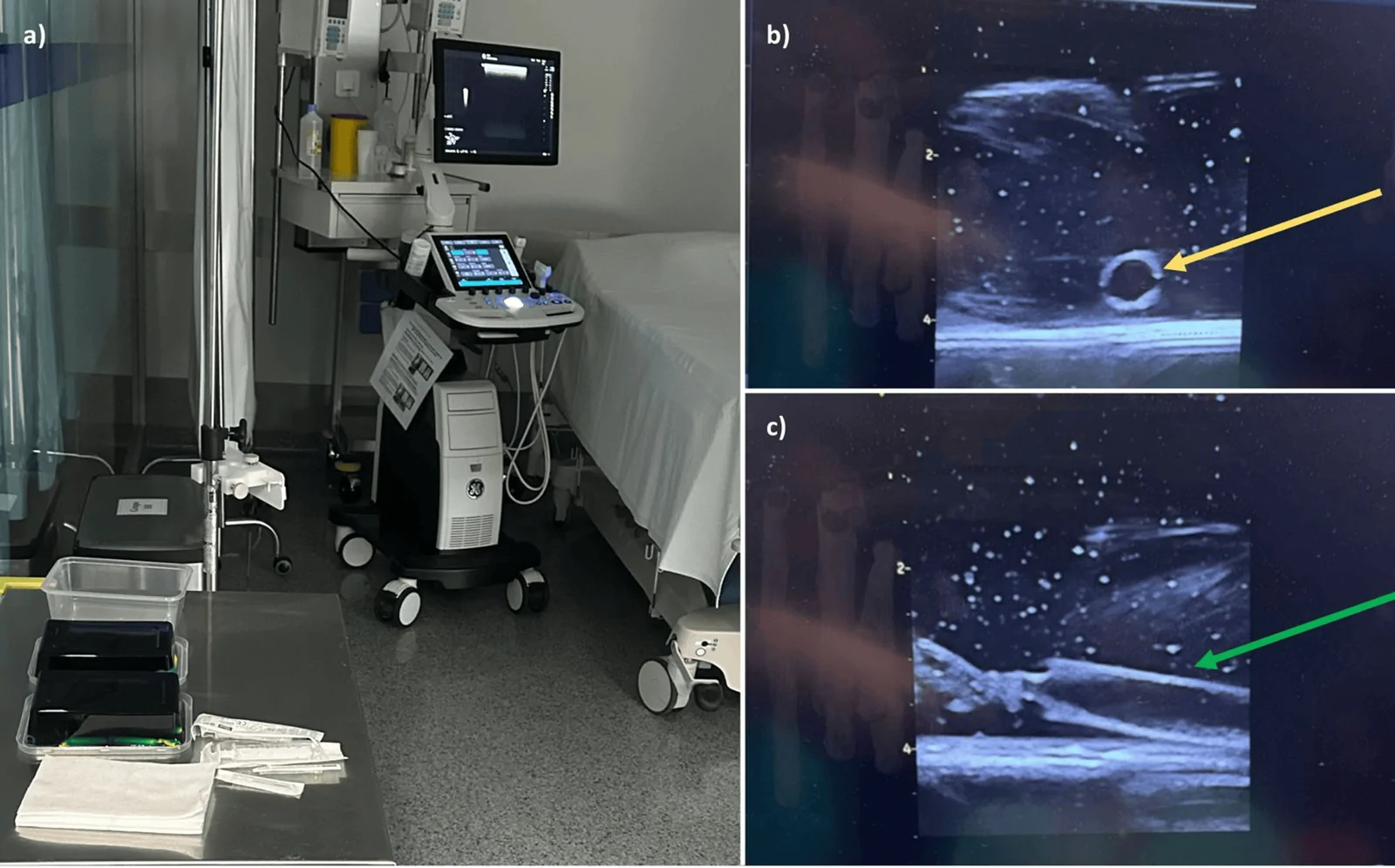
View of the homemade ultrasound phantom a) Prepared models arranged in the classroom for the practical training session, together with ultrasound machines, syringes, and needles. b) Short-axis ultrasound view of the vein (yellow arrow). c) Long-axis ultrasound view of the vein (green arrow).

The total cost to produce a set of three gelatin models was under €20. Each model allowed for approximately 12 punctures in undamaged areas, enabling each participant to perform three to four needle insertions.

Five training sessions were conducted between January 2023 and January 2025, involving all physicians and residents working in the intensive care unit during this period of time. Each session consisted of a theoretical component followed by hands-on training. Practical training included visualization of jugular and subclavian veins in healthy volunteers, followed by ultrasound-guided puncture training using the gelatin-based models, both in and out-of-plane.

Each session included between six and nine participants. Two ultrasound machines and two gelatin models were used simultaneously, with a third backup model available to ensure all participants could practice punctures on undamaged areas.

The primary outcome was the perceived utility of the session, with a secondary outcome related to the perceived value of the training model, both measured by the application of an anonymous online questionnaire that included questions about prior experience, followed by items assessing participants' perception of the training’s usefulness, as well as the quality of the instructors and training materials. Responses were measured using a 5-point Likert scale (Appendix 2).

This study was conducted as an educational quality-assessment initiative, without clinical interventions, and no identifiable personal or health information was collected. In accordance with institutional policy and national regulations applicable to educational evaluation and quality-improvement activities, formal review by a research ethics committee was not required. All participants were informed of the purpose of the evaluation and of the voluntary and anonymous nature of the questionnaire; completion and return of the questionnaire was taken as implied consent to participate. 

## Results

A total of 34 responses to the survey were collected after training a total of 34 participants. The characteristics of the respondent population are presented in Table 1. Most participants were residents enrolled in specialty training programs, with a relatively balanced distribution across Internal Medicine, Anesthesiology, and Intensive Care Medicine.

**Table 1:** Demographic and professional characteristics of training participants Data are represented as N (%).

Professional level	N (%)
Specialty residents	27 (79%)
Hospital physicians	3 (9%)
Senior hospital physicians	4 (12%)
Specialty	
Intensive care medicine	8 (24%)
Internal medicine	11 (32%)
Anesthesiology	6 (18%)
General surgery	4 (12%)
Other	5 (14%)
Experience with landmark-guided CVC placement	
Fewer than 10 procedures	24 (71%)
11-50 procedures	3 (9%)
More than 50 procedures	7 (21%)
Experience with ultrasound-guided CVC placement	
Fewer than 10 procedures	16 (47%)
11-50 procedures	11 (32%)
More than 50 procedures	7 (21%)

Many participants reported limited experience with landmark-guided CVC placement, and approximately half had performed more than 10 ultrasound-guided CVC insertions.

The overall evaluation of the training was favorable. Notably, 87% of respondents agreed or strongly agreed that the training was relevant to their clinical practice.

When asked whether the training had a positive impact on their confidence in performing ultrasound-guided CVC placement, 97% of participants reported that the gelatin model contributed positively. Similarly, 91% considered ultrasound practice on volunteers to be beneficial.

Areas with lower satisfaction ratings included the availability of ultrasound machines per trainee (3%), the availability of training models (6%), and the duration of the sessions (6%).

Questionnaire responses were analyzed descriptively only. As the study lacked a control group, no inferential statistical comparisons were performed, and the questionnaire served as a qualitative appraisal of participant satisfaction with the training program.

## Discussion

Our experience showed that gelatin-based models, which are inexpensive and easy to prepare, can be successfully integrated into training programs with positive perceived outcomes.

The vast majority of participants reported increased confidence in performing ultrasound-guided punctures after the training -- a particularly relevant finding considering that nearly half had previously performed more than 10 such procedures. This suggests that even among practitioners with some prior experience, structured training using practical models retains pedagogical value.

The most frequently mentioned limitations and suggestions were not related to the model but rather to the availability of ultrasound machines per trainee and the limited time available for practice. These findings underscore the importance of planning sessions with adequate equipment and sufficient time for supervised hands-on training.

From a practical standpoint, the models proved to be durable, easy to assemble, and capable of producing satisfactory ultrasound imaging. Compared with commercial phantoms, they represent an extremely cost-effective alternative, particularly in low-budget training environments. 

This study has some limitations, including the lack of objective assessment of training effectiveness (e.g., formal practical evaluation or through success and complication rates before and after the implementation of a training protocol using these models), and the reliance on subjective self-reported outcomes from participants. Future studies could explore the correlation between this type of training and actual clinical performance.

## Conclusions

Ultrasound-guided CVC placement is widely recommended as standard practice in modern critical care, yet the acquisition of technical proficiency remains dependent on structured and repeated training. In this context, our experience demonstrates that low-cost gelatin-based simulation models represent a feasible and highly accessible tool for training. Our models were well accepted by participants and were associated with a consistent increase in self-reported confidence, even among clinicians with prior experience in ultrasound-guided vascular access. This finding suggests that low-cost simulation may have a role not only in early training but also with experienced practitioners. 

Although this study is limited by its reliance on subjective outcomes and the absence of objective performance metrics, it provides pragmatic evidence that low-cost, easily reproducible models can be successfully integrated into structured training programs. Such approaches may be particularly valuable in resource-limited settings, where access to commercial simulators is difficult. Future research should focus on objectively evaluating skill acquisition and retention, as well as on determining whether training with gelatin-based models translates into improved clinical performance and reduced complication rates. Ultimately, integrating accessible simulation tools into formal training curricula may contribute to safer and more effective vascular access practices in critical care.

## Appendices

Appendix 1

Phantom Preparation for Ultrasound-Guided Venous Access Training

A low-cost, gelatin-based vascular phantom was developed to simulate venous structures for ultrasound-guided puncture training in critical care settings.

Materials

(1) Long, thin latex balloons (modeling balloons), (2) water (optionally tinted with red food coloring), (3) fishing line, (4) gelatin sheets (50 g total; equivalent to 5-6 packets of 9 g), (4) boiling water (800 mL), (5) dark food coloring (blue or similar), (6) rectangular plastic container, (7) weights to prevent balloon flotation (e.g., metal screw and nut assembly), and (7) refrigerator

Phantom Construction

Long, thin latex balloons were filled with water and sealed with a knot at one end. Red food coloring was optionally added to enhance visual resemblance to venous blood. Using fishing line, four to five knots were tied along the length of each balloon, creating multiple distinct segments. This allowed repeated ultrasound-guided venous access attempts within the same simulated vessel while preserving overall structural integrity.

Separately, 800 mL of freshly boiled water was prepared. Gelatin sheets (totaling 50 g) were gradually dissolved one by one in the hot water with continuous gentle stirring to avoid air incorporation. Mechanical mixing (e.g., electric whisking) was avoided, as it results in excessive foaming and ultrasound artifacts. Dark blue food coloring was added incrementally until the solution became markedly opaque, improving ultrasound contrast between the simulated vessels and the surrounding medium.

The prepared balloons were positioned at the bottom of a rectangular plastic container. To prevent flotation during pouring, weights were used to anchor the balloons in place; a large metal screw with a nut at each end, forming an “H”-shaped anchor, proved effective.

The gelatin solution was carefully poured into the container until the balloons were fully submerged. The phantom was refrigerated for at least 12 hours (overnight) to allow complete solidification. Once set, the phantom was demolded and ready for use, remaining stable for several hours at room temperature.

If balloon migration occurred during solidification, the phantom could be demolded and used in an inverted orientation, ensuring that the simulated vessels remained at an appropriate depth for ultrasound-guided puncture training.

Final Characteristics

Once solidified, the phantom provided a stable, homogeneous medium with embedded, compressible tubular structures suitable for ultrasound visualization and repeated needle puncture. The model allowed training in both short-axis and long-axis approaches for ultrasound-guided venous access.

Appendix 2

**Table 2:** Participant satisfaction questionnaire *Likert scale: 1 = Strongly disagree; 2 = Disagree; 3 = Neither agree nor disagree; 4 = Agree; 5 = Strongly agree.

Item	Question/statement	Response format
1	Current stage in medical career	General training resident (foundation year); specialty training resident; consultant; senior consultant; senior consultant with advanced grade
2	Specialty currently in training	Intensive Care Medicine; Internal Medicine; Anesthesiology; General Surgery; Other
3	Medical specialty	Intensive Care Medicine; Internal Medicine; Anesthesiology; General Surgery; Other
4	Experience in central venous catheter placement using anatomical landmarks	Fewer than 10 procedures; 11-50 procedures; more than 50 procedures
5	Experience in ultrasound-guided central venous catheter placement	Fewer than 10 procedures; 11-50 procedures; more than 50 procedures
6	The practical training in ultrasound-guided vascular access was relevant to my clinical practice.	Likert scale (1-5)*
7	The instructors provided adequate support throughout the training.	Likert scale (1-5)*
8	The instructor-to-trainee ratio was appropriate.	Likert scale (1-5)*
9	The ultrasound equipment used was adequate for the objectives of the training.	Likert scale (1-5)*
10	The ultrasound machine-to-trainee ratio was appropriate.	Likert scale (1-5)*
11	Ultrasound training performed on volunteers increased my confidence in performing ultrasound-guided vascular access in real patients.	Likert scale (1-5)*
12	Training with gelatin-based models increased my confidence in performing ultrasound-guided vascular access in real patients.	Likert scale (1-5)*
13	The gelatin-based models were appropriate for the objectives of the training.	Likert scale (1-5)*
14	The ratio of training models per trainee was appropriate.	Likert scale (1-5)*
15	The time allocated to the training session was adequate.	Likert scale (1-5)*
16	Overall, the training fulfilled its proposed objectives.	Likert scale (1-5)*
17	Please provide any comments or suggestions regarding this training.	Free-text response

## References

[1] Troianos CA, Hartman GS, Glas KE, Skubas NJ, Eberhardt RT, Walker JD, Reeves ST (2012). Guidelines for performing ultrasound guided vascular cannulation: recommendations of the American Society of Echocardiography and the Society Of Cardiovascular Anesthesiologists. Anesth Analg.

[2] Rupp SM, Apfelbaum JL, Blitt C (2012). Practice guidelines for central venous access: a report by the American Society of Anesthesiologists task force on central venous access. Anesthesiology.

[3] Lamperti M, Bodenham AR, Pittiruti M (2012). International evidence-based recommendations on ultrasound-guided vascular access. Intensive Care Med.

[4] Frankel HL, Kirkpatrick AW, Elbarbary M (2015). Guidelines for the appropriate use of bedside general and cardiac ultrasonography in the evaluation of critically ill patients-part I: general ultrasonography. Crit Care Med.

[5] Bouaziz H, Zetlaoui PJ, Pierre S (2015). Guidelines on the use of ultrasound guidance for vascular access. Anaesth Crit Care Pain Med.

[6] Franco-Sadud R, Schnobrich D, Mathews BK (2019). Recommendations on the use of ultrasound guidance for central and peripheral vascular access in adults: a position statement of the Society of Hospital Medicine. J Hosp Med.

[7] (2020). Practice guidelines for central venous access 2020: an updated report by the American Society of Anesthesiologists task force on central venous access. Anesthesiology.

[8] Lamperti M, Biasucci DG, Disma N (2020). European Society of Anaesthesiology guidelines on peri-operative use of ultrasound-guided for vascular access (PERSEUS vascular access). Eur J Anaesthesiol.

[9] Barsuk JH, Cohen ER, Feinglass J, McGaghie WC, Wayne DB (2009). Use of simulation-based education to reduce catheter-related bloodstream infections. Arch Intern Med.

[10] Carraccio C, Burke AE (2010). Beyond competencies and milestones: adding meaning through context. J Grad Med Educ.

[11] Kalivoda EJ, Sullivan A, Bunting L (2019). A cost-effective, rapidly constructed simulation model for ultrasound-guided pericardiocentesis procedural training. J Emerg Med.

[12] Sabak M, Al-Hadidi A, Demashkieh L, Zengin S, Hakmeh W (2022). Homemade phantoms improve ultrasound-guided vein cannulation confidence and procedural performance on patients. Ulus Travma Acil Cerrahi Derg.

[13] Mason MM, Richardson KD, Carino Mason MR, Swonger RM, Emami S, Anantha S, Thornton LM (2024). Two affordable, high-fidelity central venous models for ultrasound-guided interventional training. Simul Healthc.

[14] Kendall JL, Faragher JP (2007). Ultrasound-guided central venous access: a homemade phantom for simulation. Can J Emerg Med.

[15] Madsen EL, Zagzebski JA, Banjavie RA, Jutila RE (1978). Tissue mimicking materials for ultrasound phantoms. Med Phys.

[16] Culjat MO, Goldenberg D, Tewari P, Singh RS (2010). A review of tissue substitutes for ultrasound imaging. Ultrasound Med Biol.

[17] Kumar P (2017). News. J Med Phys.

[18] Di Domenico S, Santori G, Porcile E, Licausi M, Centanaro M, Valente U (2007). Inexpensive homemade models for ultrasound-guided vein cannulation training. J Clin Anesth.

[19] Rossi UG, Rigamonti P, Torcia P (2015). Congenital anomalies of superior vena cava and their implications in central venous catheterization. J Vasc Access.

[20] Amato ACM, Freitas SL, Veloso PM (2015). Gelatin model for training ultrasound-guided puncture. Jornal Vascular Brasileiro.

[21] Lui J, Vaghadia H (2017). An easily assembled phantom for teaching ultrasound-guided vascular access. Can J Anaesth.

